# Incidental pneumatosis intestinalis in a patient with chronic kidney disease: a case report

**DOI:** 10.1093/jscr/rjaf168

**Published:** 2025-03-28

**Authors:** Lina Berrada Dirhoussi, Florence Latinis

**Affiliations:** Department of Visceral Surgery, Établissement Hospitalier de Nord Vaudois (eHNV), Yverdon-les-Bains, 1400 Vaud, Switzerland; Department of Visceral Surgery, Établissement Hospitalier de Nord Vaudois (eHNV), Yverdon-les-Bains, 1400 Vaud, Switzerland

**Keywords:** pneumatosis intestinalis, chronic kidney disease, hemodialysis computed tomography, conservative management

## Abstract

Pneumatosis intestinalis (PI) is a clinical condition characterized by the presence of gas within the bowel wall and is considered a rare medical finding. While PI may be harmless in some cases, it can also signal more severe gastrointestinal pathology, including ischemia, bowel obstruction, or perforation. We present the case of an 87 year old male with end-stage kidney disease undergoing hemodialysis (HD), in whom PI was incidentally discovered during a follow up computed tomography (CT) performed for a renal mass. The patient was asymptomatic, and the CT findings showed no signs of ischemia, bowel obstruction or perforation. This case underscores the importance of correlating clinical and imaging data to avoid unnecessary surgery interventions. Additionally, it provides a comprehensive review of the pathogenesis, causes, diagnosis and management of PI. Further research is warranted to investigate the potential link between chronic kidney disease, HD, and the development of PI.

## Introduction

Pneumatosis intestinalis (PI) is an uncommon radiological finding with a wide spectrum of clinical implications, ranging from benign to life-threatening. Although its pathogenesis is multifactorial, PI is broadly categorized into primary (idiopathic) and secondary forms, with the latter linked to underlying gastrointestinal (GI) or systemic conditions [1, 2]. This report presents an asymptomatic case of PI discovered incidentally in a patient undergoing a follow-up computed tomography (CT) for a renal mass. The absence of symptoms or complications allowed for conservative management (CM). This case provides an opportunity to explore the existing literature on PI, emphasizing its diagnostic and therapeutic challenges.

## Clinical case

An 87-year-old man with end-stage chronic kidney disease (CKD) secondary to probable hypertensive nephroangiosclerosis (stage G5A3) was undergoing follow-up CT imaging for a renal mass. He had a history of arteriovenous fistula formation in the left upper arm for hemodialysis (HD) in 2020 and later on cephalic vein angioplasty in 2022. He had no other significant medical history.

A CT scan performed for acute urinary retention revealed a 16-mm exophytic mass in the lower pole of the right kidney, infiltrating adjacent fatty tissues, and a 1-cm inter-aortocaval lymph node. Both kidneys appeared atrophic with simple cortical cysts.

**Figure 1:**
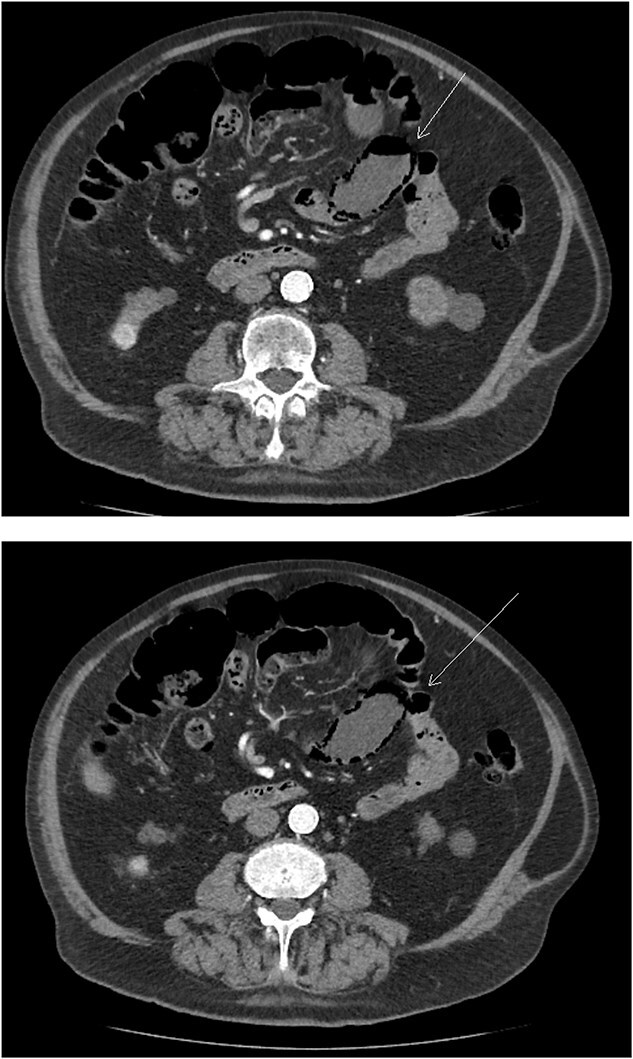
Axial abdominal CT scan image where the arrow points to the presence of pneumatosis intestinalis.

A follow-up CT three months later showed a slight increase in the kidney lesion size but no evidence of surface or distant metastasis. Notably, PI of the small bowel was observed (Fig. 1) but there were no findings of bowel ischemia (BI) or obstruction, such as mesenteric ischemia (MI), bowel wall thickening, ascites, or pneumoperitoneum.

The patient remained asymptomatic throughout the observation period, and no intervention was required.

## Discussion

PI can be primary or secondary, with the idiopathic form being rare and benign, while secondary PI is more common and associated with underlying GI conditions or systemic risk factors (RF). Approximately 15% of cases are primary, asymptomatic, and self-limiting. Most cases (85%) are secondary, linked to conditions such as intestinal ischemia, infections (Clostridia or *Escherichia coli*), blunt trauma, COPD, endoscopic procedures, and immunosuppressive therapy. Secondary PI has a higher risk of complications such as intestinal perforation, ischemia, or obstruction, making timely medical or surgical intervention critical [3].

PI affects the colon (40%–46%), followed by the small intestine (27%), stomach (5%), and both intestines in 7% of the cases [4, 5]. The colonic form is more often benign, while small bowel PI has a greater risk of ischemia [6]. In this case, the patient exhibited no systemic signs, associated RF, or radiological complications, reinforcing the diagnosis of primary PI.

The pathogenesis of PI is multifactorial, with overlapping theories:

- **Mechanical Theory:** Gas infiltrated the bowel wall due to increased intraluminal pressure, often from blunt trauma, endoscopy or obstruction
- **Bacterial Theory:** Gas-producing bacteria, such as *Echerichia coli*, invade the bowel wall, releasing gas into the submucosa.
- **Pulmonary Theory:** In patients with pulmonary conditions such as obstructive pulmonary disease (COPD), alveolar rupture allows gas to travel through mesenteric vessels and into the bowel wall.

These mechanisms can coexist, explaining cases like this one, where HD- induced stress and bacterial translocation may contribute to PI [3, 7, 8].

PI is rare, with a prevalence estimated at 0.03% of the population. The true rate may be higher due to undiagnosed asymptomatic cases [4, 9]. It predominantly affects males (3:1 ratio) and is more common in individuals aged 50–80 years [5, 10]. Many cases are asymptomatic; however, symptoms such as abdominal pain, bloating, diarrhea, or nausea may occur. In severe cases, PI can lead to bowel ischemia, perforation, or septic shock [10]. Lassandro *et al*. [1] emphasize the importance of radiological findings, in diagnosing PI, as symptoms alone cannot predict the condition.

RF associated with severe outcomes include advanced age (≥60 years), metabolic acidosis, leukocytosis, acute abdomen on physical examination, hypotension and acute kidney injury. Imaging findings such as bowel wall thickening, ascites, of portal venous gas (PVG), or bowel dilatation on CT scan [6]. This patient’s absence of the aforementioned RF, along with his stable clinical presentation and normal laboratory values, further supported a CM.

CT is the gold standard for diagnosing PI. Findings include bowel wall gas and, in severe cases, PVG. Additional CT indicators, such as bowel wall thickening, MI, or pneumoperitoneum, help identify high-risk cases requiring urgent intervention [4]. In this patient, the absence of these features and his asymptomatic presentation strongly suggested a benign form of PI.

Although many cases of PI are benign, severe cases may lead to ischemia, perforation, peritonitis, or sepsis. Mortality rates range from 50% to 75% in complicated PI, particularly those involving mesenteric ischemia. Predictors of severe outcomes include hypotension, metabolic acidosis, elevated white cell count, and PVG. Early recognition and differentiation between benign and life-threatening PI are crucial for appropriate management [3, 10].

Treatment depends on the underlying cause and clinical severity. Asymptomatic or benign cases, like this one, are best managed conservatively. Observation and monitoring are often sufficient [7]. Treatment options include:

- **Oxygen Therapy:** High-flow or hyperbaric oxygen promotes gas resorption and inhibits anaerobic bacterial growth.
- **Antibiotics:** Empirical use of metronidazole and quinolones is effective in suspected bacterial overgrowth.
- **Endoscopic Interventions:** Cyst aspiration is reserved for obstruction of significant symptoms
- **Surgical Management:** Required only for complications such as perforation or ischemia [6, 11].

This case highlights the importance of recognizing incidental PI and differentiating it from life-threatening conditions. The absence of systemic signs and radiological complications in this patient supported conservative management. Identifying risk factors and complications is key to optimizing strategies. Further studies are needed to better understand the associations between CKD, HD, and PI development.
